# Immediate Implant Placement and Provisionalization Without Guided Bone Regeneration or Connective Tissue Grafting: A Case Report

**DOI:** 10.7759/cureus.94469

**Published:** 2025-10-13

**Authors:** Nobuhiro Katahira, Hana Takegawa, Sumihiko NIkaido, Keisuke Seki

**Affiliations:** 1 Private Practice, Katahira Dental Office, Tokyo, JPN; 2 Implant Dentistry, Nihon University School of Dentistry Dental Hospital, Tokyo, JPN

**Keywords:** bone regeneration, esthetic zone, guided surgery, immediate implant placement, provisionalization

## Abstract

Immediate implant placement in the maxillary esthetic zone remains a challenging procedure, requiring careful case selection and precise technique to achieve optimal esthetic and functional outcomes. This case report describes the successful immediate placement of an implant (Straumann BLX, 3.75 mm diameter, 12.0 mm length; Institut Straumann AG, Basel, Switzerland) in the maxillary right central incisor position following the atraumatic extraction of a vertically fractured tooth. A fully guided surgical approach was used without flap elevation or bone grafting. Immediate provisionalization was performed using a screw-retained provisional crown designed to support soft tissue healing and maintain esthetic outcome. At the six-month follow-up, clinical and radiographic examinations demonstrated successful osseointegration, healthy peri-implant soft tissue, and spontaneous buccal bone regeneration without guided bone regeneration. The provisional restoration maintained excellent esthetic outcomes, with stable gingival contours and no complications. This case demonstrates that immediate implant placement and provisionalization in the maxillary esthetic zone can be successfully performed without bone grafting when appropriate case selection criteria are met, including intact buccal bone walls and adequate primary stability.

## Introduction

Immediate implant placement following tooth extraction is a well-established treatment modality in contemporary implant dentistry [1-3]. This approach offers several advantages, including reduced treatment time, preservation of alveolar bone dimensions, and improved patient comfort by avoiding multiple surgical procedures [4,5]. However, immediate implant placement in the maxillary esthetic zone presents unique challenges because of the critical importance of maintaining optimal esthetic outcomes and the complex anatomy of the anterior maxilla [6-8]. Preoperative guided bone regeneration (GBR) and postoperative connective tissue grafting (CTG) are treatment options for achieving esthetic success in the maxillary anterior region [9-11]. However, these hard and soft tissue grafts pose challenges such as risk of infection and significant invasiveness. The success of immediate implant placement depends on several key factors, including adequate bone volume for primary stability, an intact buccal bone wall, absence of acute infection, and proper three-dimensional implant positioning [12,13]. The International Team for Implantology (ITI) SAC classification system for case selection and risk assessment in immediate implant treatment has been established [14], providing valuable guidance for clinical decision-making [15]. Furthermore, it has been reported that successful treatment requires appropriate provisionalization after immediate implant placement following tooth extraction [16,17].

This case report describes the successful esthetic management of a vertically fractured maxillary central incisor using immediate implant placement and provisionalization without bone grafting, demonstrating the potential for predictable outcomes when appropriate protocols are followed.

## Case presentation

Initial presentation and diagnosis

**Figure 1:**
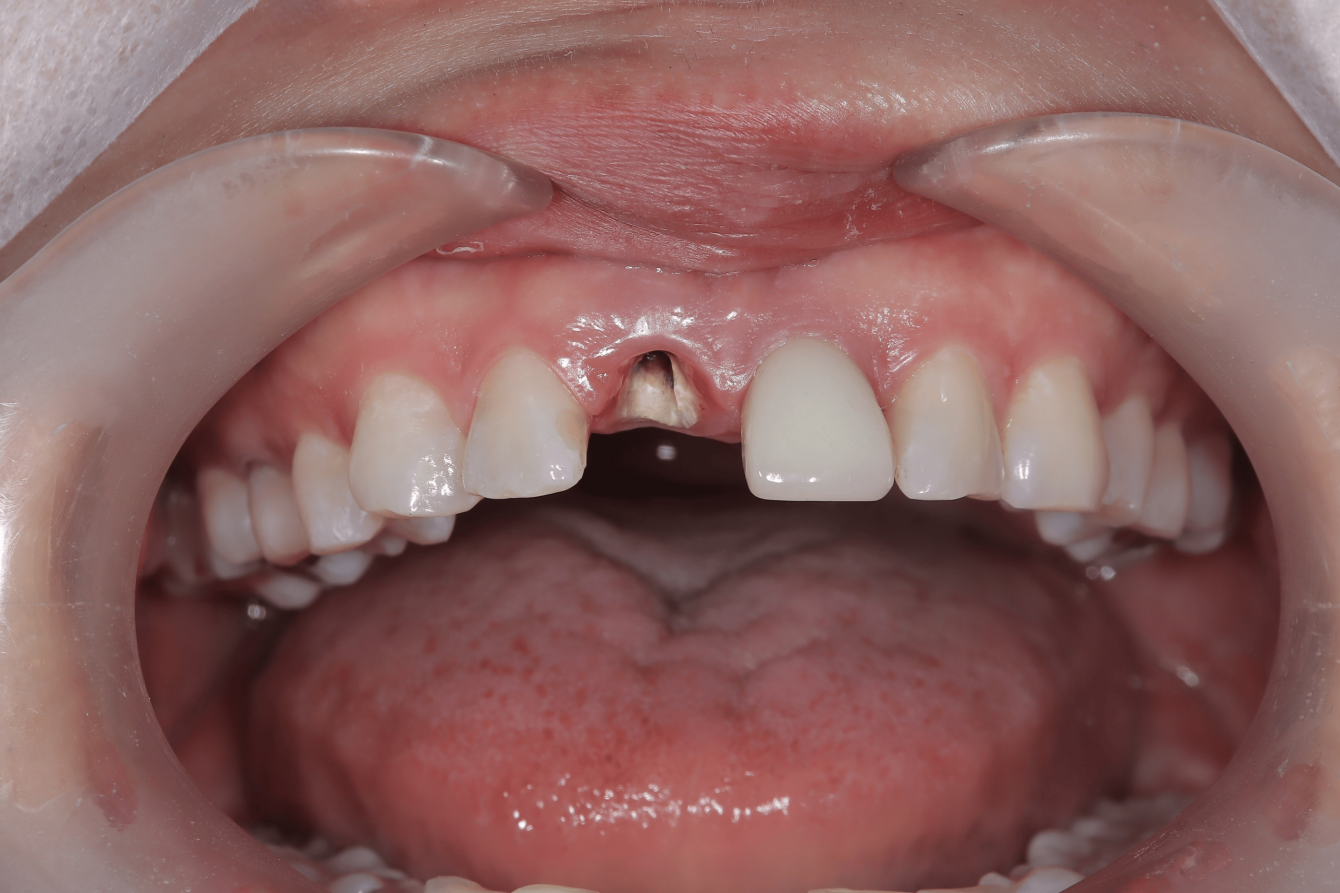
Preoperative clinical view Preoperative clinical view of the maxillary right central incisor (#11) with extensive coronal destruction.

**Figure 2:**
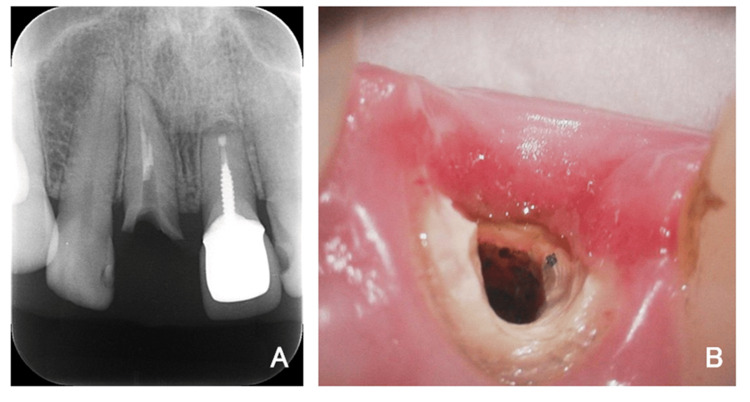
Root fracture of the maxillary right central incisor (A) Periapical radiograph showing the root fracture of the tooth. (B) Intraoral close-up image of the maxillary right central incisor (#11) showing a vertical root fracture with subgingival exposure. Inflammation of the marginal gingiva and a compromised ferrule effect are evident.

A 50-year-old female patient presented with extensive coronal destruction of the maxillary right central incisor (#11) caused by a vertical root fracture (Figure 1). Clinical examination revealed subgingival exposure of the fracture line, associated marginal gingival inflammation, and a compromised ferrule effect (Figure 2).

Digital treatment planning

**Figure 3:**
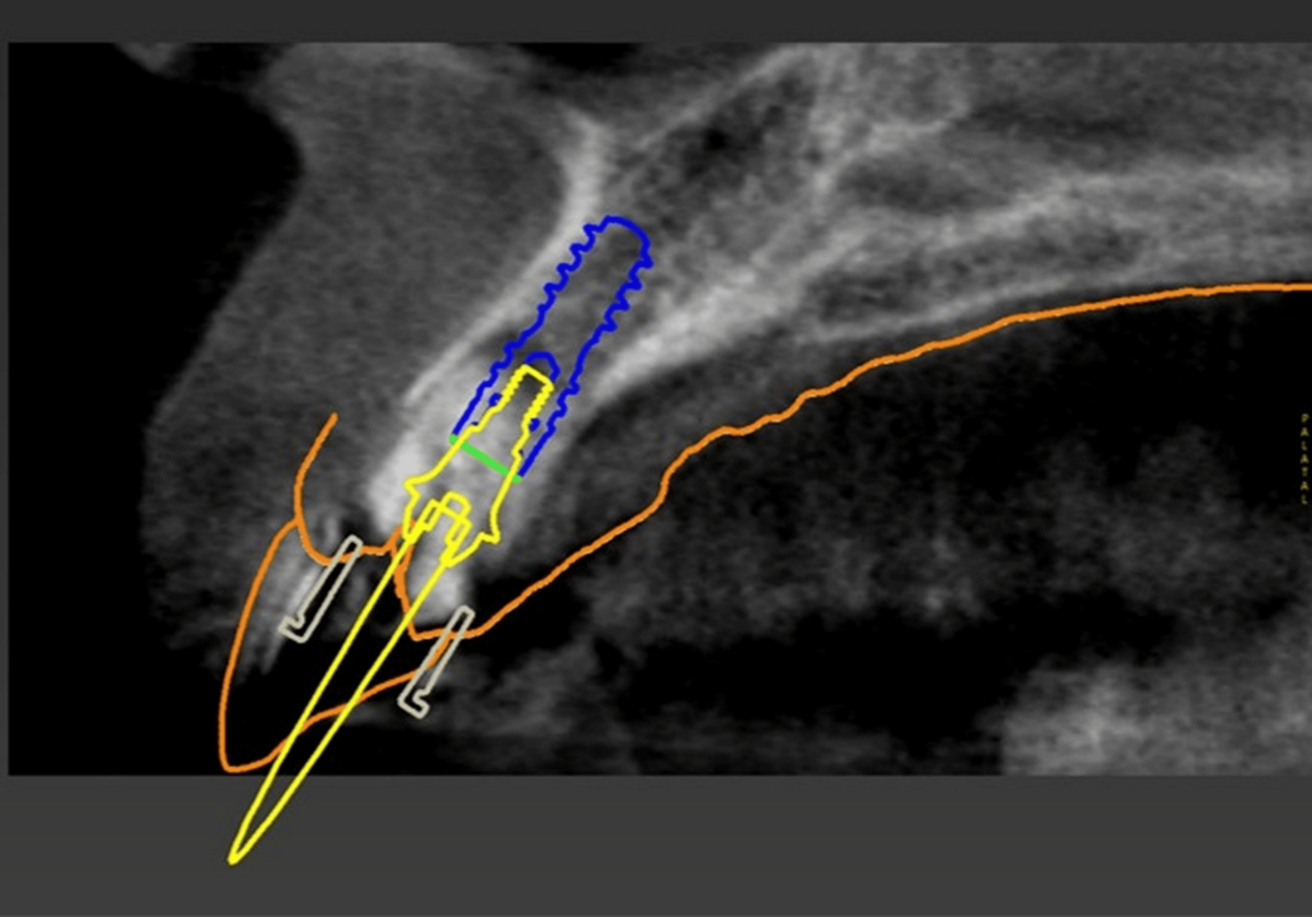
Virtual planning cone-beam computed tomography (CBCT) image Cross-sectional CBCT image showing virtual planning for immediate implant placement. A Straumann BLX implant (diameter 3.75 mm, length 12.0 mm) was positioned palatally within the socket using a guided surgical approach. A screw-retained abutment (SRA) is planned to support immediate provisionalization. The thickness of the labial bundle bone was 1.5 mm, and it was diagnosed as healthy. The anticipated peri-implant gap was approximately 2 mm, allowing natural healing without bone grafting.

**Figure 4:**
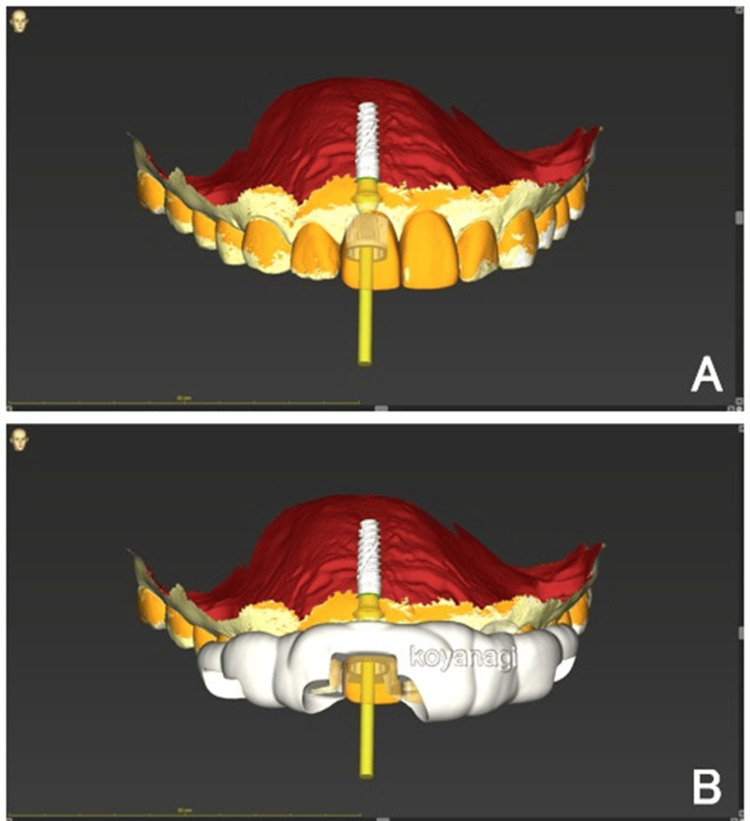
Preoperative prosthetic digital wax-up (A) Digital model showing preoperative prosthetic wax-up merged with intraoral scan and soft-tissue data. (B) Design of the full-arch 3D-printed surgical guide and the prefabricated provisional crown based on cone-beam computed tomography (CBCT) and intraoral scan alignment. This fully guided approach allows precise, flapless implant placement and immediate provisionalization according to the planned emergence profile.

Comprehensive digital planning was performed using CBCT imaging and intraoral scanning. Virtual implant placement was planned for an implant (Straumann BLX, 3.75 mm diameter, 12.0 mm length; Institut Straumann AG, Basel, Switzerland) positioned palatally within the extraction socket. The anticipated peri-implant gap was approximately 2 mm, which falls within the acceptable range for natural healing without bone grafting, according to International Team for Implantology consensus statements (Figure 3). The preoperative digital wax-up and surgical guide designs are shown in Figure 4.

Surgical procedure

**Figure 5:**
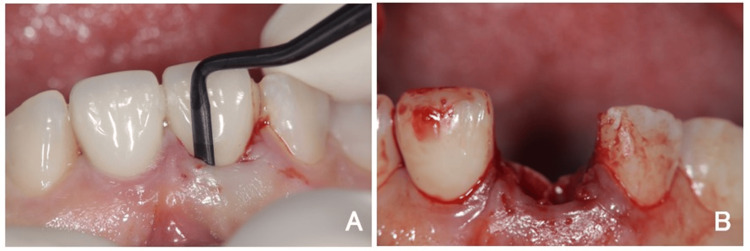
Tooth extraction performed using a periotome (A) The initial tooth extraction step was performed using a periotome. The instrument was gently inserted into the periodontal ligament space to minimize trauma to the surrounding alveolar bone and soft tissues. A flapless approach was used throughout this study. (B) Clinical view of the extraction socket following the removal of the fractured incisor. All bone walls were intact, and the buccal plate was preserved. Flap elevation or grafting was not performed at this stage.

A periotome was used for atraumatic extraction to minimize trauma to the surrounding alveolar bone and soft tissues (Figure 5). A flapless approach was maintained throughout the procedure to preserve the blood supply and soft tissue architecture.

Guided implant placement

**Figure 6:**
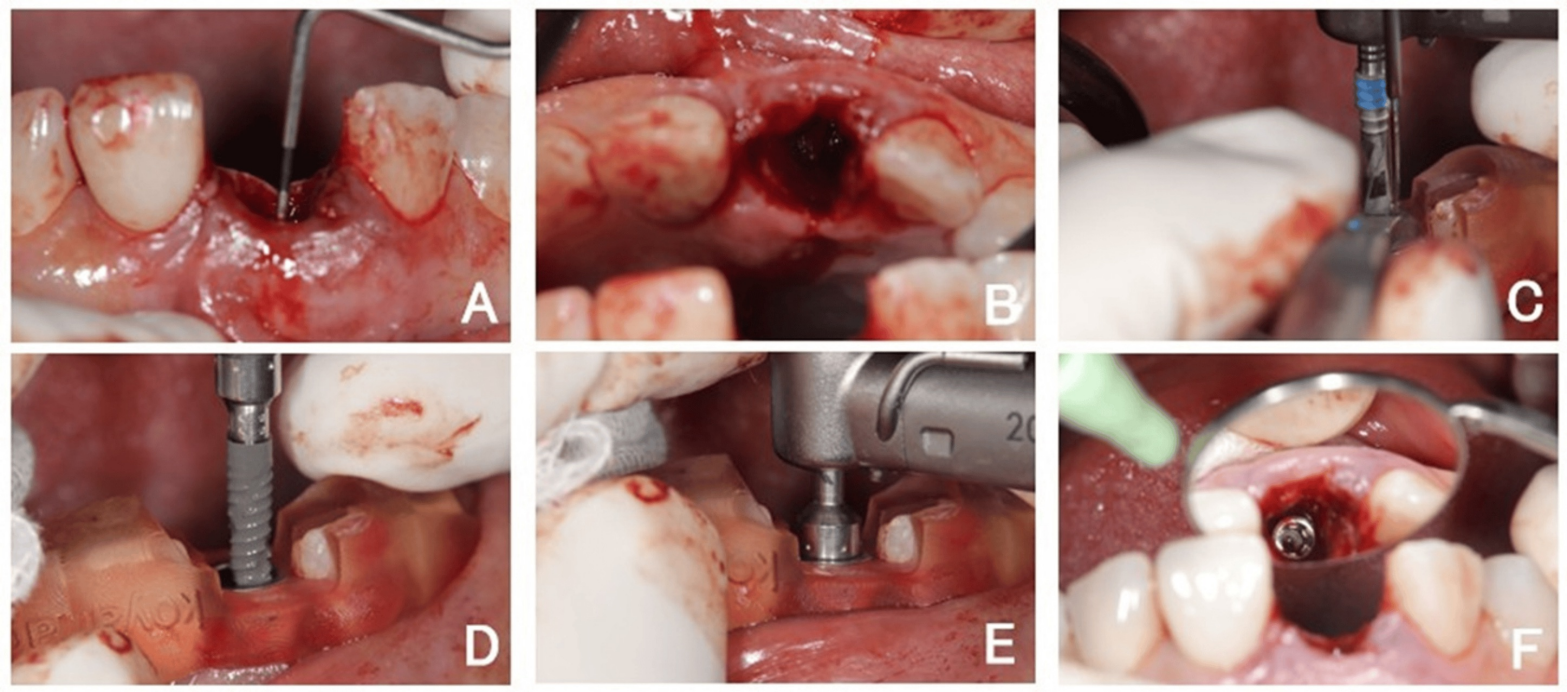
Guided implant insertion in a flapless procedure Clinical sequence illustrating guided implant osteotomy and insertion in a flapless procedure. (A) Post-extraction probing confirmed intact socket and buccal bone wall. (B) Occlusal view of the fresh socket. (C-E) Sequential osteotomy drilling using a full-arch surgical guide. (F) Final seating of the Straumann BLX implant, achieving high primary stability (~35–45 N cm), placed slightly palatal for screw-retained restoration.

Sequential drilling was performed using a surgical template to achieve ideal three-dimensional positioning (Figure 6). The esthetic superstructure was planned with the access hole positioned on the palatal side. The implant was placed slightly palatal to allow for screw-retained restoration and to achieve high primary stability (35-45 N·cm). Implant stability was measured using an analytical device (Osseo100+, NAKANISHI Inc., Tochigi, Japan) during surgery, and the implant stability quotient value was 73.

Immediate provisionalization

**Figure 7:**
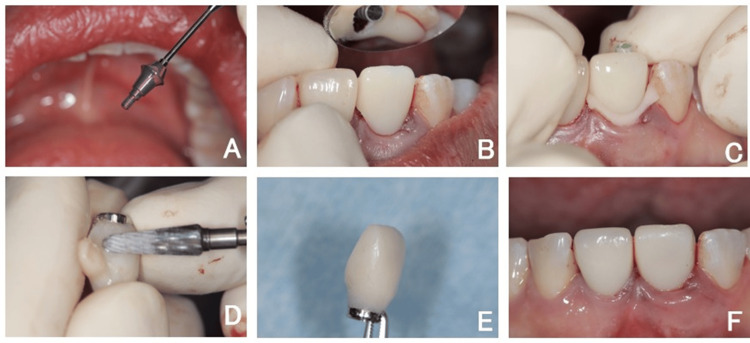
Immediate provisionalization (A) Clinical sequence showing immediate provisionalization using a screw-retained abutment (SRA) in a one-abutment one-time protocol. (B-D) The provisional crown was seated without buccal pressure, adjusted extraorally, and placed out of occlusion to support soft tissue healing. (E, F) The technique was designed to maintain circulation in the buccal soft tissues.

A screw-retained provisional restoration was carefully adjusted and seated to prevent compression of the buccal tissue while achieving a proper emergence profile. The restoration was designed to support soft tissue contouring and guide natural gingival healing (Figure 7).

Healing and follow-up

**Figure 8:**
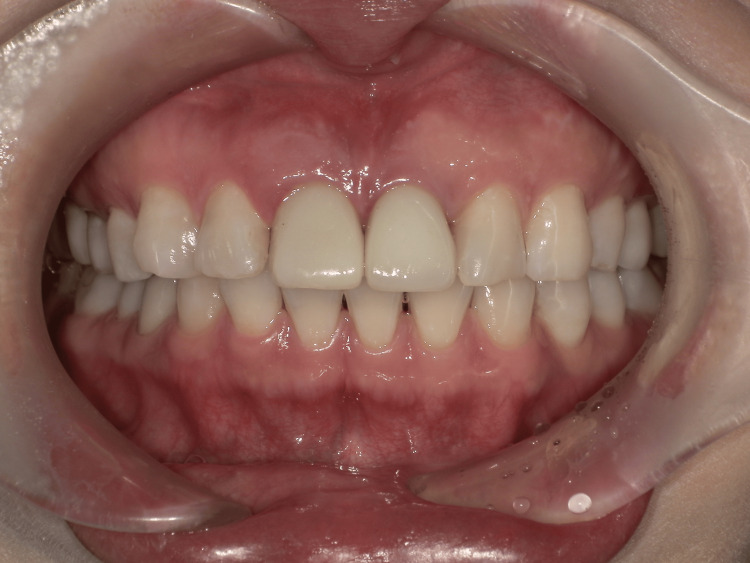
Provisional restoration Intraoral view after provisional restoration delivery.

Progressive soft tissue healing and maturation were observed during sequential follow-up appointments (Figure 8). Intraoral photographs taken one week after placement of the provisional restoration showed no signs of inflammation in the marginal gingiva, which appeared naturally pink. There was no loss of interdental papillae or gingival recession, and the scalloped contour harmonized well with the adjacent tooth.

**Figure 9:**
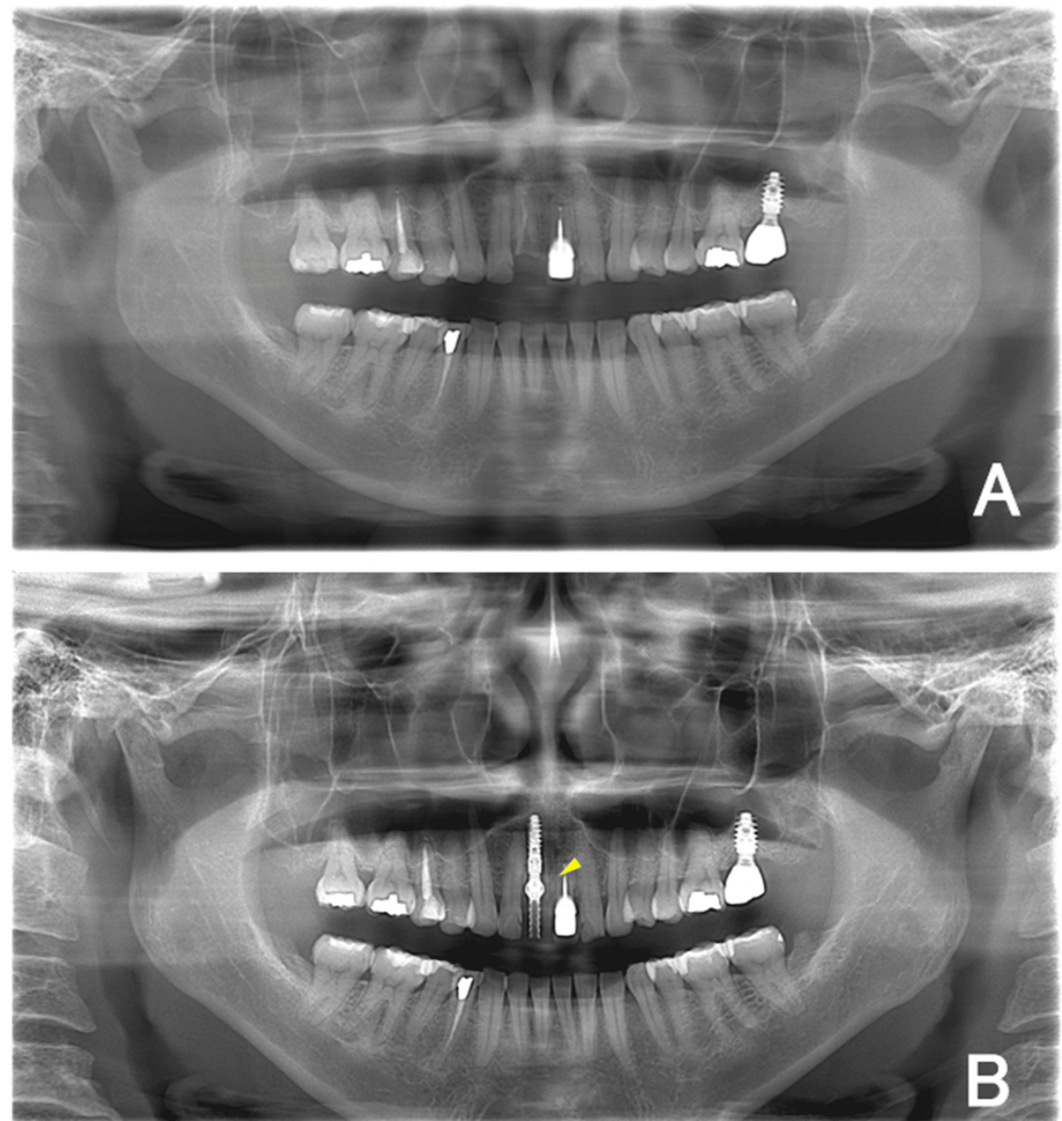
Panoramic radiographs before and after implant treatment (A) Preoperative image showing the fractured maxillary right central incisor (#11). (B) Postoperative view demonstrating the correct 3D positioning of the implant, stable crestal bone levels, and absence of complication (arrowhead).

Radiographic evaluation performed one month later revealed maintaining healthy bone levels without the use of guided bone regeneration (Figure 9).

Final restoration

**Figure 10:**
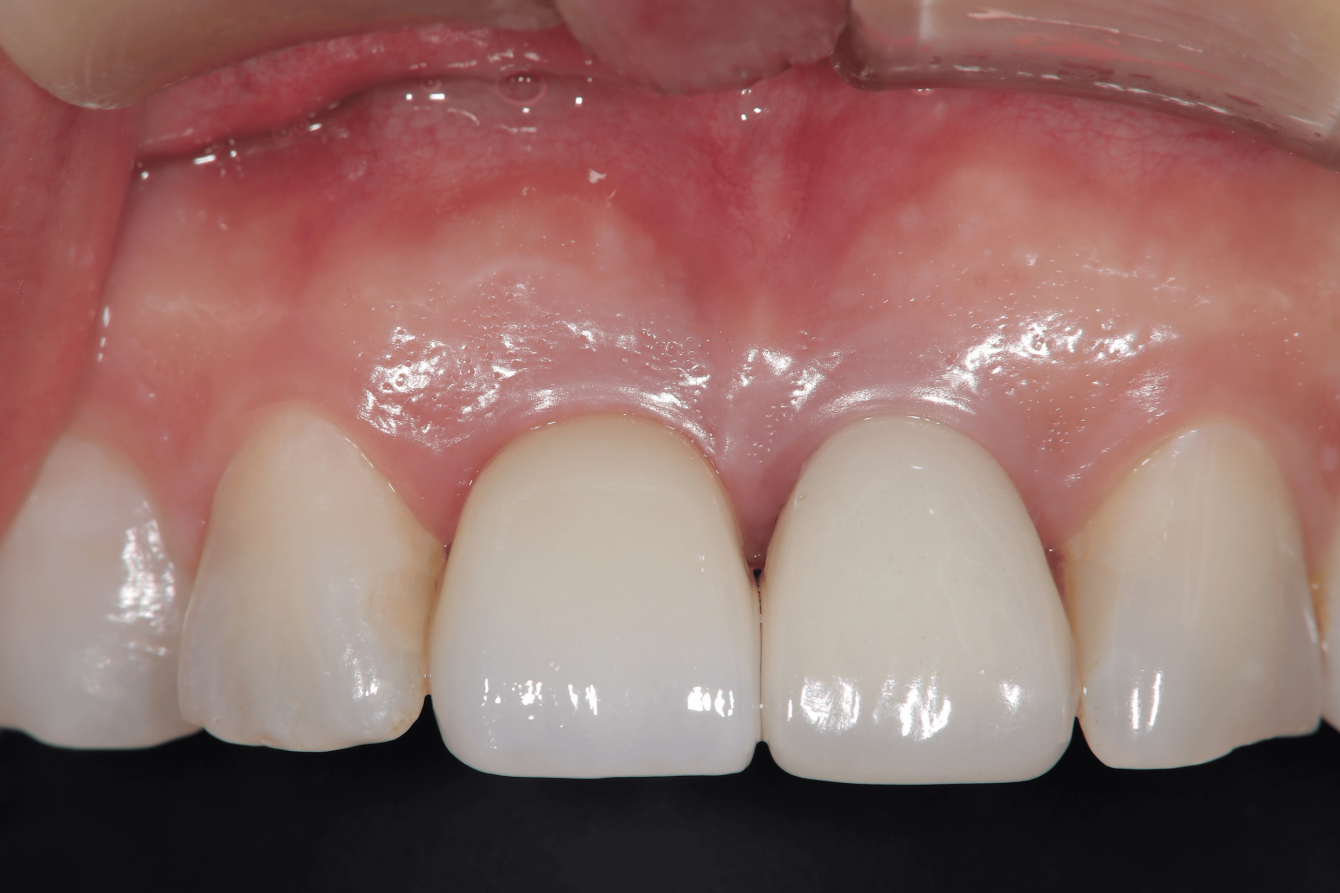
Postoperative clinical view Intraoral view after final restoration delivery (three months after placing provisional restoration).

The screw-retained crown on implant #11 showed harmonious color, contour, and gingival integration with the adjacent natural teeth (Figure 10).

## Discussion

This case demonstrates the successful application of immediate implant placement and provisionalization in the maxillary esthetic zone, without bone grafting (Table 1).

**Table 1:** Comparison items and key advantages

Comparison Item	BLT + GBR + CTG (Conventional Two-Stage)	BLX + Immediate Provisionalization (Current Case)	Key Advantages
Surgical technique	Flap elevation + GBR + CTG, two-stage approach	Flapless, no graft, one-stage approach	Minimally invasive, shorter duration
Soft tissue handling	CTG to augment tissue volume	Preserved blood supply + SRA-guided contouring	Stable without grafting
Bone management	GBR (Bio-Oss^®^ + autogenous bone)	No graft; natural healing in < 2 mm gap	Smaller field, biologically friendly
Implant system	Straumann^®^ BLT (good primary stability)	Straumann^®^ BLX (high torque capacity)	Evolved primary stability
Prosthetic protocol	Two-stage: temporary → final	Immediate temp → provisional → final	Fewer visits, less patient stress
Esthetics	Maintained with graft and design	Supported by perfusion and provisional	Sufficient tissue stability
Treatment duration	~4-6 months or more	~2-3 months	Significantly shortened

Several key factors contributed to the favorable outcomes achieved in this case. Atraumatic extraction using a periotome is crucial for preserving the buccal bone wall and maintaining the architecture of the extraction socket. This approach aligns with current evidence supporting the importance of socket preservation in achieving immediate implant success [18,19]. The fully guided surgical approach allowed for precise three-dimensional implant positioning, ensuring optimal placement for both functional and esthetic outcomes.

The palatal positioning of the implant facilitated the use of a screw-retained restoration, which offers advantages in terms of retrievability and maintenance in the esthetic zone. The observation of spontaneous buccal bone regeneration at the one-month follow-up without guided bone regeneration is consistent with ITI consensus statements, indicating that peri-implant gaps ≤2 mm in intact sockets may heal predictably without augmentation procedures. This finding supports the hypothesis that proper case selection and surgical technique can minimize the need for additional bone grafting [20].

The immediate provisionalization protocol, designed to avoid buccal tissue compression while supporting soft tissue contouring, contributed to the excellent soft tissue outcomes observed throughout the healing period. The emergence profile was carefully designed to guide natural gingival healing and to maintain papilla formation.

This case represents a straightforward (S) classification according to the ITI SAC Assessment, given the intact buccal bone wall, adequate bone volume, and absence of active infection [15]. The predictable outcomes support the use of immediate implant placement in appropriately selected cases. The five key factors for success include careful case selection with intact buccal bone walls, atraumatic extraction technique, precise guided implant placement, appropriate immediate provisionalization, and maintenance of a flapless surgical approach.

One limitation of this study was the absence of postoperative CBCT evaluation. Only two-dimensional imaging was performed to reduce patient radiation exposure. However, CBCT is optimal for evaluating three-dimensional bone volume and should be utilized in future cases. The second limitation is the absence of long-term clinical observations regarding soft tissue stability. Further research with a larger case series and longer follow-up periods would be valuable to validate these findings and establish predictable protocols for immediate implant placement in the esthetic zone.

## Conclusions

This case report demonstrates that immediate implant placement and provisionalization in the maxillary esthetic zone can achieve excellent clinical and esthetic outcomes when appropriate case selection criteria are met. The combination of atraumatic extraction, guided implant placement, and immediate provisionalization without bone grafting resulted in successful osseointegration, spontaneous bone regeneration, and stable soft tissue outcomes.
